# Vascular Endothelial Expression of Indoleamine 2,3-Dioxygenase 1 Forms a Positive Gradient towards the Feto-Maternal Interface

**DOI:** 10.1371/journal.pone.0021774

**Published:** 2011-07-06

**Authors:** Astrid Blaschitz, Martin Gauster, Dietmar Fuchs, Ingrid Lang, Petra Maschke, Daniela Ulrich, Eva Karpf, Osamu Takikawa, Michael G. Schimek, Gottfried Dohr, Peter Sedlmayr

**Affiliations:** 1 Institute of Cell Biology, Histology and Embryology, Center for Molecular Medicine, Medical University of Graz, Graz, Austria; 2 Division of Biological Chemistry, Biocenter, Innsbruck Medical University, Innsbruck, Austria; 3 Department of Obstetrics and Gynecology, Medical University of Graz, Graz, Austria; 4 Institute of Pathology, Center for Applied Biomedicine, Medical University of Graz, Graz, Austria; 5 Laboratory of Radiation Safety, National Institute of Longevity Science, National Center for Geriatrics and Gerontology, Obu City, Japan; 6 Institute for Medical Informatics, Statistics and Documentation, Medical University of Graz, Graz, Austria; Université Paris Descartes, France

## Abstract

We describe the distribution of indoleamine 2,3-dioxygenase 1 (IDO1) in vascular endothelium of human first-trimester and term placenta. Expression of IDO1 protein on the fetal side of the interface extended from almost exclusively sub-trophoblastic capillaries in first-trimester placenta to a nearly general presence on villous vascular endothelia at term, including also most bigger vessels such as villous arteries and veins of stem villi and vessels of the chorionic plate. Umbilical cord vessels were generally negative for IDO1 protein. In the fetal part of the placenta positivity for IDO1 was restricted to vascular endothelium, which did not co-express HLA-DR. This finding paralleled detectability of IDO1 mRNA in first trimester and term tissue and a high increase in the kynurenine to tryptophan ratio in chorionic villous tissue from first trimester to term placenta. Endothelial cells isolated from the chorionic plate of term placenta expressed IDO1 mRNA in contrast to endothelial cells originating from human umbilical vein, iliac vein or aorta. In first trimester decidua we found endothelium of arteries rather than veins expressing IDO1, which was complementory to expression of HLA-DR. An estimation of IDO activity on the basis of the ratio of kynurenine and tryptophan in blood taken from vessels of the chorionic plate of term placenta indicated far higher values than those found in the peripheral blood of adults. Thus, a gradient of vascular endothelial IDO1 expression is present at both sides of the feto-maternal interface.

## Introduction

Pregnancy implicates a state of peaceful coexistence between hemiallogeneic tissues of the mother and the fetus. Interest in the role of the tryptophan-degrading enzyme indoleamine 2,3-dioxygenase in the context of feto-maternal tolerance and in immunosuppression in general was aroused more than ten years ago [1], [2]. While placental expression of indoleamine 2,3-dioxygenase (IDO) may not necessarily be a prerequisite for the tolerance-mediating role of the enzyme, most studies have focused on investigations of the direct cellular interfaces between mother and fetus, which is located in the decidua basalis, where the fetally derived invading trophoblast may be recognized and tolerated by the maternal uterine immune system [3] and the huge surface of syncytiotrophoblast which covers the placental villous trees and separates the maternal and fetal blood circulations from each other. So a number of efforts have been undertaken for localization of the enzyme in the placenta. Initially IDO expression at the feto-maternal interface was described in glandular epithelial cells of uterine glands, trophoblast cells and macrophages [4], [5], [6], [7], but also endothelial cells might express IDO [6], [7] however, not all of the studies came to the same immunolocalization results. IDO expression in dendritic cells of tumor-draining lymph nodes [8] prompted an unsuccessful search for the same phenomenon in the regional lymph nodes of uteri of pregnant mice (P. Arck, A. Blaschitz, P. Sedlmayr; unpublished observations). Other than mediating immunosuppression, IDO displays antimicrobial and antiviral effects by reducing the availability of the essential amino acid tryptophan in the inflammatory environment [9], [10].

Further enzymes catalyzing the same step in tryptophan catabolizm may also be expressed in the placenta. This has been shown for tryptophan-dioxygenase (TDO) in the mouse, where expression of TDO precedes expression of IDO [11]. TDO displays low sequence similarity to IDO and in contrast to IDO is not blocked by 1-methyl tryptophan [12]. Recently indoleamine 2,3-dioxygenase 2 (IDO2) with 43% identity at amino acid level to IDO (henceforth named IDO1) has been characterized. It is also expressed in the placenta and is preferentially inhibited by 1-methyl-D-tryptophan, in contrast to preferential inhibition of IDO1 by the L-enantiomer. The tryptophan-degrading activity of IDO2 is probably much lower compared to IDO1, the biological role as yet unclear [13], [14], [15], [16], [17].

Vascular endothelial cells (EC) have been implicated in expression of IDO1 and tryptophan-degrading activity in the context of infectious diseases, tumour pathology and transplantation [18]. Taking into account the particular immunological situation of the utero-placental unit, it was of special interest to investigate IDO1 expression and activity with special regard to vascular endothelial cells on both sides of the feto-maternal interface.

In the present study we investigated paraffin-embedded placenta and decidua tissues from early and term gestational stages and various anatomical locations using an improved immunohistochemical protocol. Furthermore we measured the enzyme activities of sera and tissues and identified IDO1 mRNA in isolated endothelial cells from the placenta and other organs.

## Materials and Methods

### Ethics Statement

The present study was approved by the Ethics Committee of the Medical University of Graz, Austria (No. 20-074 ex 08/09 and No. 19-293 ex 07/08). The Ethics Committee of the Medical University of Graz (Ethikkommission der Medizinischen Universität Graz, http://www.meduni-graz.at/ethikkommission/Graz/) is registered at the Office for Human Research Protections (OHRP) of the US Department of Health and Human Services (DHHS) with the number IRB00002556. Written informed consent was obtained from the blood and tissue donors involved in the study. In addition, samples taken from the paraffin block archive of the Institute of Cell Biology, Histology and Embryology in Graz were used. Use of these archival samples was approved by the Ethics Committee of the Medical University of Graz as of March 19, 1999 and September 11, 2003, without the condition of obtaining written consent.

### Tissues and cells

Tissue and blood samples from 10 normal term placentas were collected after spontaneous delivery or caesarian section and 10 samples from first trimester pregnancy were obtained after pregnancy terminations between the 6^th^ and the 11^th^ week. Furthermore, the following archival samples of interest for this study were used: three samples of hysterectomy post partum (one due to placenta increta, two due to postpartal atony), a sample of a uterus in the 22^nd^ week of pregnancy from a case of uterine rupture and a sample of abdominal skin of a pregnant woman had been excised for a scar from a previous caesarian section during the course of a second section (39^th^ week). For the purpose of comparison of concentrations of tryptophan and kynurenine we used a set of data established previously from the peripheral blood of 38 healthy blood donors [19].

Placental endothelial cells (PEC) were isolated from placental tissue as described previously [20]. Endothelial cells from isolated V. iliacae originating from adult donors were harvested by enzymatic digestion of the inner surface of the blood vessel with Dulbeccós modified minimal essential medium (DMEM; PAA) containing 200 U/ml collagenase (type II; Sigma, Taufkirchen, Germany), trypsin inhibitor (type II S; 1 mg/ml; Sigma), calcium chloride (0.5 m; Roth), essential and non-essential amino acids (0.02 v/v; PAA), vitamins (0.01 v/v; PAA), bovine serum albumin (BSA; 2 mg/ml; Sigma), 5% fetal calf serum (FCS; PAA) and antibiotics. Digestion was allowed to proceed at 37°C for 60 min before flushing the collagenase solution containing the endothelial cells. The cell suspension was collected in FCS and centrifuged at 200 x *g* for 5 min. The pellet thus obtained was resuspended in EGM-MV medium (Lonza, Verviers, Belgium) and the cells were plated on culture plates pre-coated with 1% gelatin (Sigma). The endothelial identity of the isolated cells was confirmed by staining for the classical endothelial marker von Willebrand factor and by measuring of DiI-Ac-LDL uptake. In general, ≥99% cells in the primary culture expressed these endothelial cell characteristics. Human umbilical vein endothelial cells (HUVEC) were purchased from PromoCell (Heidelberg, Germany) and human aortic endothelial cells (HAEC) were obtained from Lonza. All endothelial cell types were cultured in EGM-MV medium (Lonza) including the supplement kit EGM-MV SingleQuots (Lonza) in humidified atmosphere containing 5% CO_2_ at 37°C.

### Antibodies

We used a mouse monoclonal anti-IDO1 antibody (IgG1) at a final concentration of 1 µg/ml [21]. For identifying extra-villous trophoblasts we utilized an antibody against HLA-G (BD Pharmingen, clone 4H84, mouse IgG1, 1 µg/ml) [22], [23]. An antibody against the epitope class II of CD34 (Dako, clone QBEnd10, mouse IgG, 0.05 µg/ml) was employed for staining endothelial cells in serial sections. Anti-HLA-DR was obtained from Becton-Dickinson (Schwechat, Austria) and used at a dilution of 1∶50. Anti-CD163 (IgG1, clone 5C6-FAT, Acris, Hiddenhausen, Germany) was applied as a macrophage marker. For negative controls we used an IgG1 isotype control antibody (Dako) at 1 µg/ml.

### Immunohistochemistry

For immunolocalisation only paraffin sections were used. Tissues were fixed between 24 h and 3 d in 4% buffered formalin at room temperature and embedded in paraffin following a standard over-night dehydration and paraffin infiltration protocol. For staining of some serial sections with anti-HLA-DR and anti-IDO1 we used HOPE-fixed paraffin sections [24]. 5 µm thick sections were cut on a sledge microtome and dried on a 50°C hot plate over night.

Sections were labelled with the anti-IDO antibody following heat induced antigen retrieval with at pH 9 (Epitope Retrieval Solution pH 9, Eubio, Vienna, Austria). This was done following optimization of the retrieval protocol where we labelled in comparison some slides after pre-treatment with citrate buffer at pH 6 or without any pretreatment. Before staining the slides were cooked under pressure in the respective buffer at 120°C for 7 min, let cool down for 20 min and washed in distilled water. The detection protocol started with a 12 min step of blocking endogenous peroxidase (Hydrogen Peroxide Block, LabVision, Fremont, CA) followed by a 7 min protein block (included in the Ultravision LP Detection system) and a 45 min step of primary antibody incubation. Bound antibodies were detected using a polyvalent (mouse, rabbit) horseradish-peroxidase polymer system (Ultravision LP Detection system, LabVision, Fremont, CA) and a 10 minute amino-ethyl-carbazole (AEC) incubation step. All slides were counter-stained with Mayer's hemalum. Washing steps were done using TBS/0.05%Tween 20. For HLA-DR labelling of HOPE sections the antigen retrieval step was not necessary and therefore skipped.

### Measurement of tryptophan and kynurenine

9 different samples of umbilical cord blood were collected shortly after delivery, sera were separated from blood cells after clotting and frozen until examination.

For collection of sera from chorionic plate arteries and veins the umbilical cord vessels were clamped and the placentae were placed directly on ice. The amnion was removed and a cannula was inserted into a chorionic artery directed to the umbilical artery to collect the arterial blood. Another cannula was introduced into a chorionic vein in the direction of the umbilical vein to harvest venous blood. As endothelium of both arteries and veins were found to express IDO1, these samples were combined into one group for statistical analysis.

In some instances plasma was used instead of sera. Earlier studies had shown no differences for the analysis of tryptophan and kynurenine between serum and plasma (D. Fuchs, unpublished results).

Chorionic villous tissues from term and first trimester placentas were balanced and frozen. Tissue material was suspended in 0.5 ml 5 mM DTE (7.71 mg/10 ml), immediately vortex mixed and then kept on a shaker for 15 min. The resulting suspension was centrifuged for 10 min at room temperature at 13,000 x *g*. For the determination of kynurenine and tryptophan concentrations HPLC with the internal calibrator 3-nitro-L-tyrosine was applied [25]. To precipitate protein 100 µl culture supernatants or serum specimens were treated with 50 µL 2 mol/L trichloroacetic acid and centrifuged. Chromatography was performed on reversed-phase cartridges LiChroCART RP_18_ columns, tryptophan was monitored via its fluorescence at 285 nm excitation and 365 nm emission wavelengths, kynurenine was measured by its UV absorption at 360 nm wavelength. The kynurenine to tryptophan ratio (kyn/trp) was calculated and expressed as µmol kynurenine/mmol tryptophan, kyn/trp allowing an estimate of IDO activity [25].

### RNA isolation of placental tissue and endothelial cells

Total RNA from placental tissues and endothelial cells was isolated with Tri Reagent (Molecular Research Center, Cincinnati, OH, USA) according to the manufacturer's instructions. In brief, small slices of frozen placenta tissue (200 mg) were homogenized in 1 ml Tri Reagent before further processing. RNA from endothelial cells was isolated by directly lysing the cells with Tri Reagent in the culture dishes.

### Semiquantitative RT-PCR

For semi-quantitative RT-PCR a commercially available RT-PCR Kit (OneStep RT-PCR Kit, Qiagen, Hilden, Germany) was used according to the manufacturer's manual. Each RT-PCR reaction contained 100 ng total RNA from tissue or primary cells. Primers were designed to get PCR products spanning exon-exon boundaries of mRNAs and had the following sequences: b-actin (GACTACCTCATGAAGATC and GATCCACATCTGCTGGAA; product 513 bp) and IDO (GGCACACGCTATGGAAAACT and CGGACATCTCCATGACCTTT; product 297 bp). The RT-PCR program included an initial 30 min step at 50°C for reverse transcription and a second 15 min step at 95°C to activate DNA polymerase. The following amplification was performed by a 3-step cycling (30 sec at 94°C, 30 sec at 60°C and 1 min at 72°C) using 24 cycles for β-actin and 32 cycles for IDO1. The PCR products were separated on 1.5% (w/v) agarose gels (SeaKem LE Agarose, Cambrex) and were documented using the FluorChemQ (Alpha Innotech) documentation system.

### Statistics

The statistical analysis of differences in concentrations in three blood compartments was performed with StatXact (a statistical package for exact nonparametric inference, part of the Cytel Studio 9 software). Mean rank differences were tested against a two-sided alternative with the Wilcoxon-Mann-Whitney-test. Each of the three parameters (tryptophan, kynurenine and their ratio) was separately compared across the three compartiments (comprising independent samples). Applying Kruskal-Wallis, we tested against an unordered alternative (there is no evidence for a certain ordering of the compartiments) under the distributional assumption that the samples have the same shape but their medians are shifted with respect to each other. Because of the small sample sizes of two compartiments, in addition to the usual asymptotic result we calculated the exact result of the comparisons.

## Results

### Localization of IDO1 protein

#### First trimester placenta

**Figure 1:**
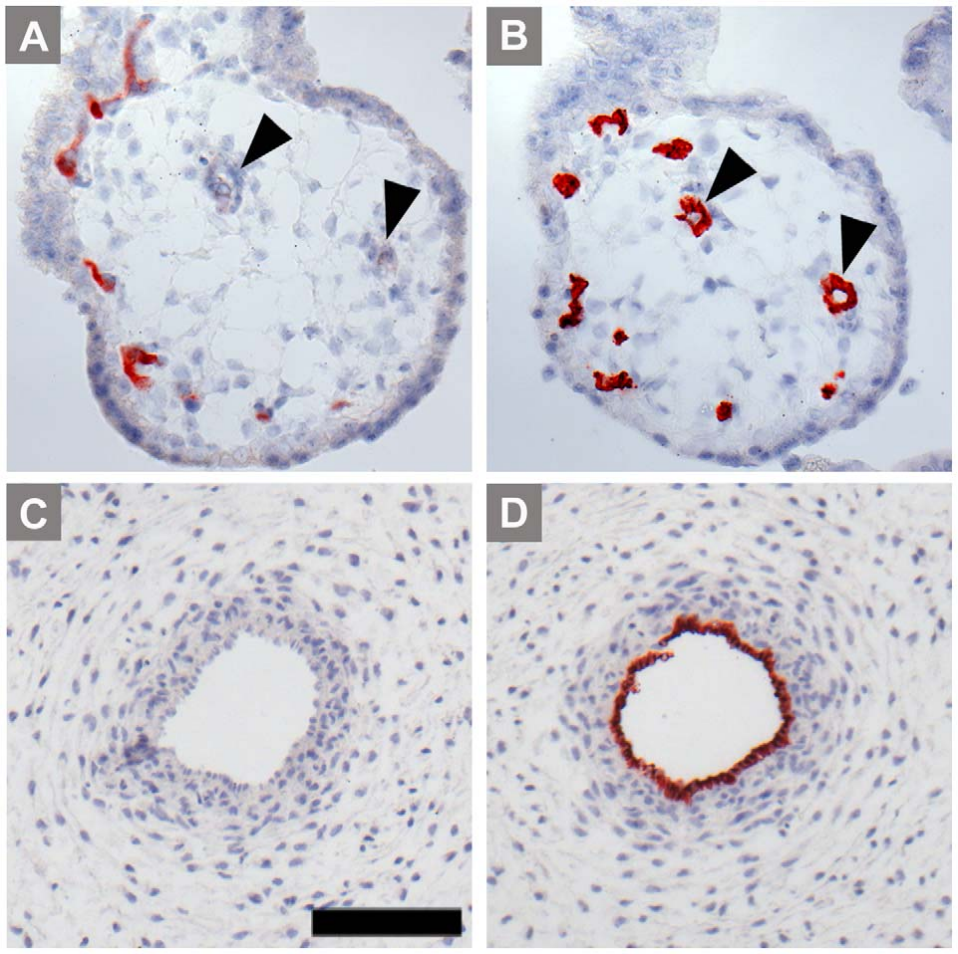
Immunohistochemical staining of first trimester placenta serial paraffin sections at the 11^th^ week of pregnancy. Villous chorion (A, B) and umbilical cord vessels (C, D) stained for IDO1 (A, C) and for the endothelial marker CD34 (B, D); bigger vessels located more to the center of the villi are indicated by arrowheads, capillaries forming a network beneath the trophoblastic double layer stain positive for IDO1. The scale bar represents 100 µm.

In villous chorion of tissue samples collected between the 6^th^ and the 11^th^ week of gestation expression of IDO1 was in most cases restricted to capillaries immediately below the trophoblastic layer (Fig.1a). Bigger vessels in the inner mesenchymal core, located at more distance from the maternal blood, were negative for IDO1, which was also absent from umbilical cord vessels (Fig. 1c). Trophoblast cells were generally negative.

**Figure 2:**
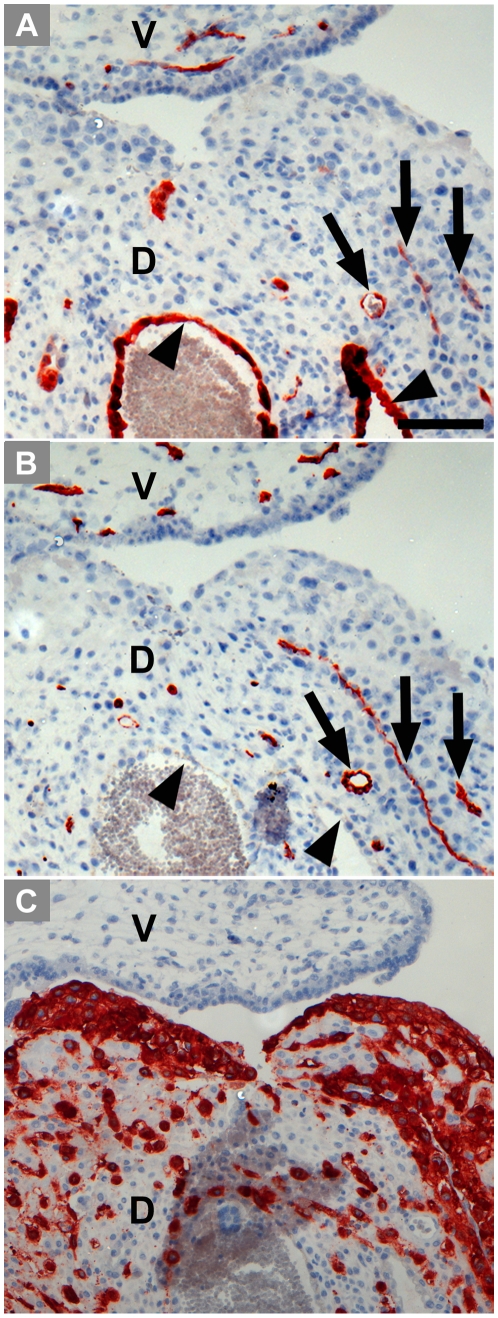
Immunohistochemical staining of serial paraffin sections of first trimester decidua basalis (D) showing also partly villous tissue (V). The sections are stained for IDO1 (A), the endothelial marker CD34 (B) and HLA–G, a marker for extravillous trophoblast (C). Arrow heads heads point towards the epithelium of uterine glands, some endothelia are indicated by arrows. The scale bar represents 100 µm.

**Figure 3:**
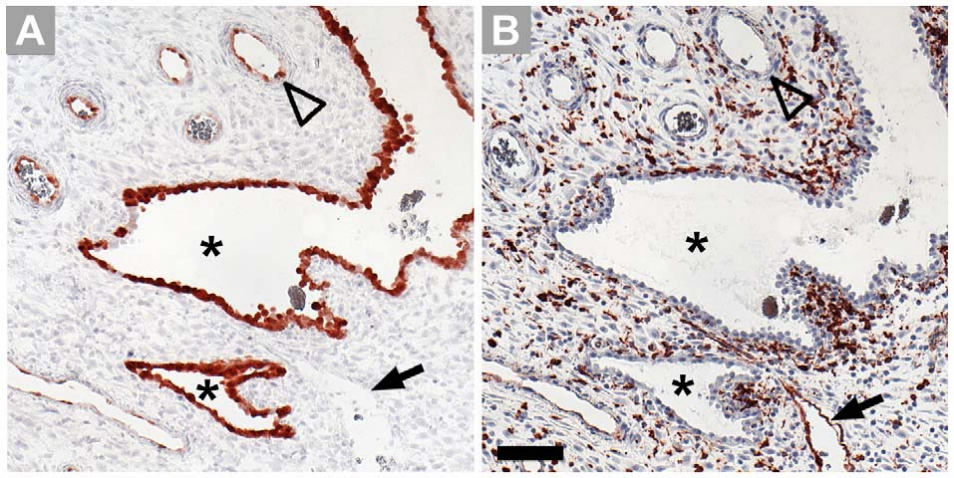
Comparison of localization of IDO1 (A) and HLA-DR (B) in first trimester decidua. Serial paraffin sections are stained by immunohistochemistry. Designated are uterine glands (asterisks), the endothelium of spiral arteries (open arrowhead) and of veins (arrow). Please note that apart from expression in the endothelium of veins, HLA-DR is expressed by macrophages. Scale bar = 100 µm.

Expression of IDO1 in decidua basalis was found in glandular epithelial cells as well as in vascular endothelial cells (Fig. 2a). Invasive HLA-G positive extravillous trophoblasts (Fig. 2c) did not stain for IDO1 in the tissue samples of decidua tested. Endothelial expression of IDO1 did not co-localize with HLA-DR, as studied in four tissue samples: Whereas IDO1 was detected predominantly in arteries, HLA-DR was found mainly in veins (Fig. 3a, b).

#### Term placenta

**Figure 4:**
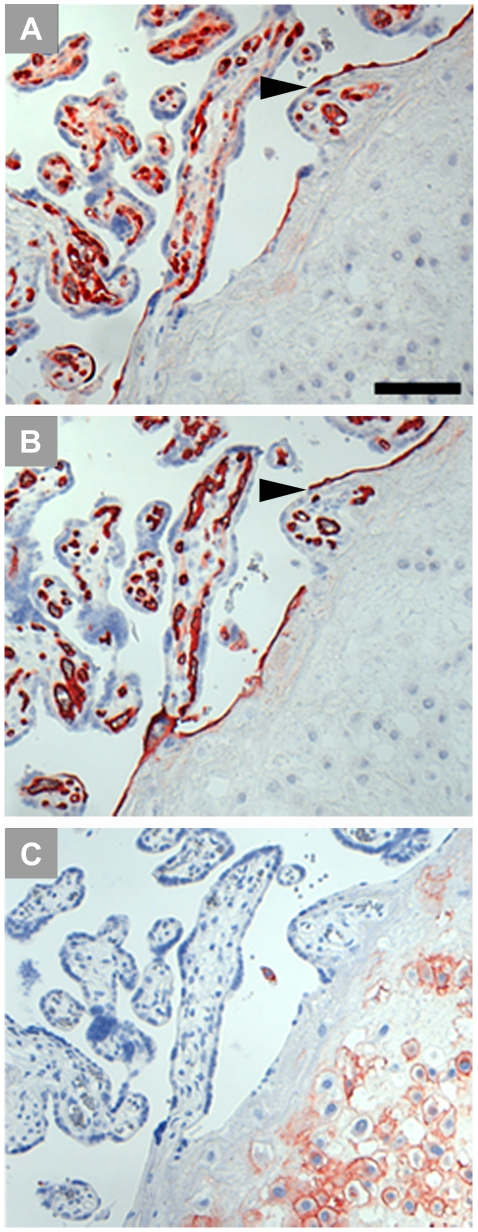
Immunohistochemical staining of term placenta serial sections including the basal plate. IDO1 labelling results in staining of vascular endothelium, leaving villous and extravillous trophoblast unstained (A). The anti-CD34 antibody stains endothelial cells of the fetal circulation in the villous mesenchymal core and also endothelial cells which line the intervillous space; the arrowhead indicates a special site where endothelium connects with syncytiotrophoblast (B). HLA-G identifies extravillous cytotrophoblasts located in the basal plate (C). Scale bar represents 100 µm.

**Figure 5:**
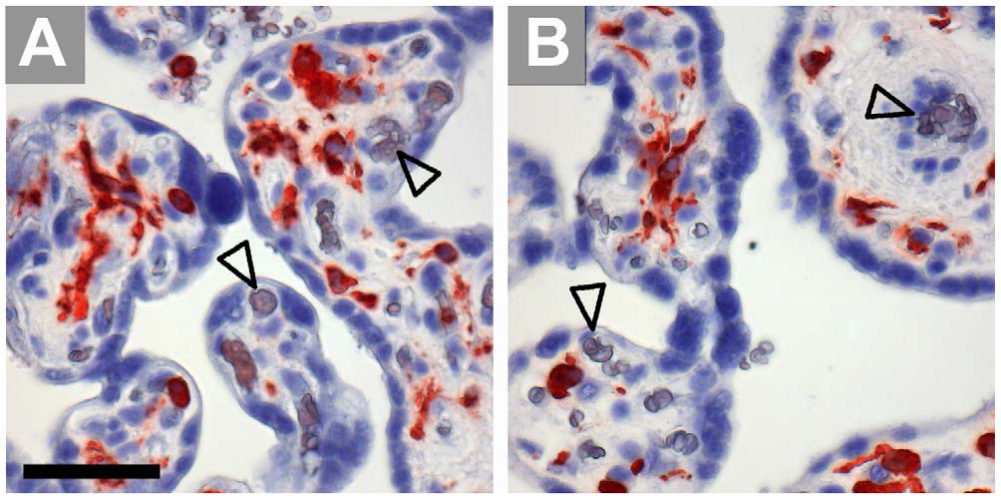
Immunohistochemical staining of paraffin sections of term villous tissue. Expression of HLA-DR (A) is allocated to Hofbauer cells, as demonstrated in a serial section stained for the macrophage marker CD163 (B), open arrowheads indicate vascular endothelia. Scale bars = 50 µm.

**Figure 6:**
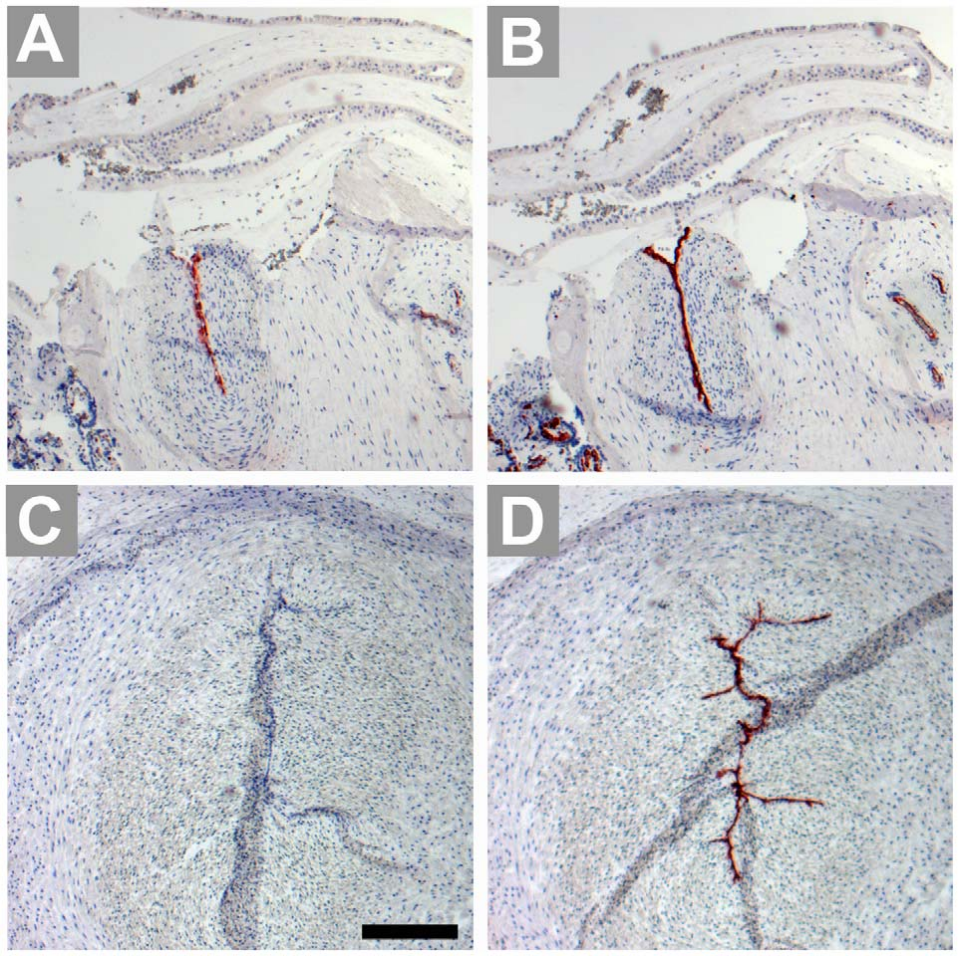
Immunohistochemical staining of serial paraffin sections of term placenta chorionic plate (A, B) and umbilical cord (C, D). The sections are stained for IDO1 (A, C) and the endothelial cell marker CD34 (B, D).Scale bar represents 100 µm.

At term, the intensity of immunohistochemical staining was found markedly increased in comparison to first trimester villi. Most endothelia of the fetal blood vessels are stained intensely for IDO1 (Fig. 4a), including also bigger vessels such as arteries and veins of stem villi. Trophoblast cells were generally negative. In the basal plate we identified cells lining the intervillous space as being endothelial cells (Fig. 4b); this layer expressed IDO1 as demonstrated in serial sections (Fig. 4). The extravillous trophoblast in term basal plate was found negative (Fig. 4). In contrast to IDO1 expression, HLA-DR was absent from vascular endothelium of small as well as of bigger vessels of chorionic villi and present on Hofbauer cells only, as found in five tissue samples (Fig. 5). Bigger vessels in the chorionic plate stained moderately for IDO1 (Fig. 6a) in most but not all of the placenta tissue samples studied. None of the ten placentas tested expressed IDO1 in vascular endothelia of the umbilical cord (Fig. 6c).

**Figure 7:**
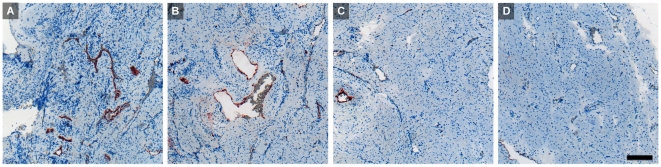
Paraffin sections of a uterine wall including the decidua in the 22^nd^ week of gestation, stained with anti-IDO1 antibody. The trophoblast-invaded implantation site shows strong staining of vascular endothelial cells (A). In the second quarter of the uterine wall most of the myometrial blood vessels are also labeled (B). The third quarter shows only one vessel stained (C) and in the outermost quarter of the uterus (D) none of the vessels stain for IDO1. Scale bar represents 200 µm.

Investigation of archival tissue paraffin blocks from a 22^nd^ week pregnancy, containing decidua and the whole uterine wall, revealed decreasing intensity of endothelial IDO1 staining towards the outer myometrium (Fig. 7). Similar results were obtained by analyzing uterine tissues post partum from three patients. One sample of a uterine artery and one of abdominal skin of a pregnant woman did not stain for IDO1 either (data not shown), indicating that maternal vessel endothelia located at a distance to the feto-maternal interface do not express IDO1**.**

### Expression of IDO1 mRNA in placenta tissues and isolated endothelial cells

**Figure 8:**
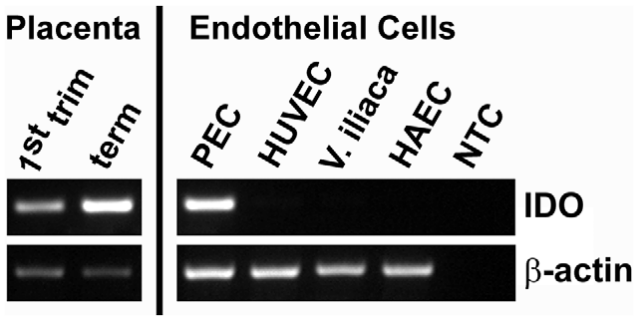
Expression of IDO1 mRNA. Semi-quantitative RT-PCR comparing IDO1 mRNA expression in first trimester and term placenta tissues with endothelial cells isolated from term placenta chorionic plate (PEC), a human umbilical vein endothelial cell line (HUVEC), endothelial cells isolated from the iliac vein (V. iliaca) and a cell line from human aortic endothelium (HAEC). The expression of beta-actin was used as internal reference and water instead of total RNA served as non template control (NTC).

The results of RT-PCR from placenta tissues indicated a marked increase in mRNA for IDO1 from first trimester to term (Fig. 8). In addition, we analyzed freshly isolated endothelia from chorionic plate veins and arteries. We compared the results to other types of endothelial cells such as the human umblical cord vein cell line HUVEC, freshly isolated cells from the human vena iliaca of the human aorta. IDO1 was detected in freshly isolated endothelial cells from the chorionic plate, whereas the other endothelia were negative (Fig. 8).

### Chorionic tryptophan degrading activity

**Figure 9:**
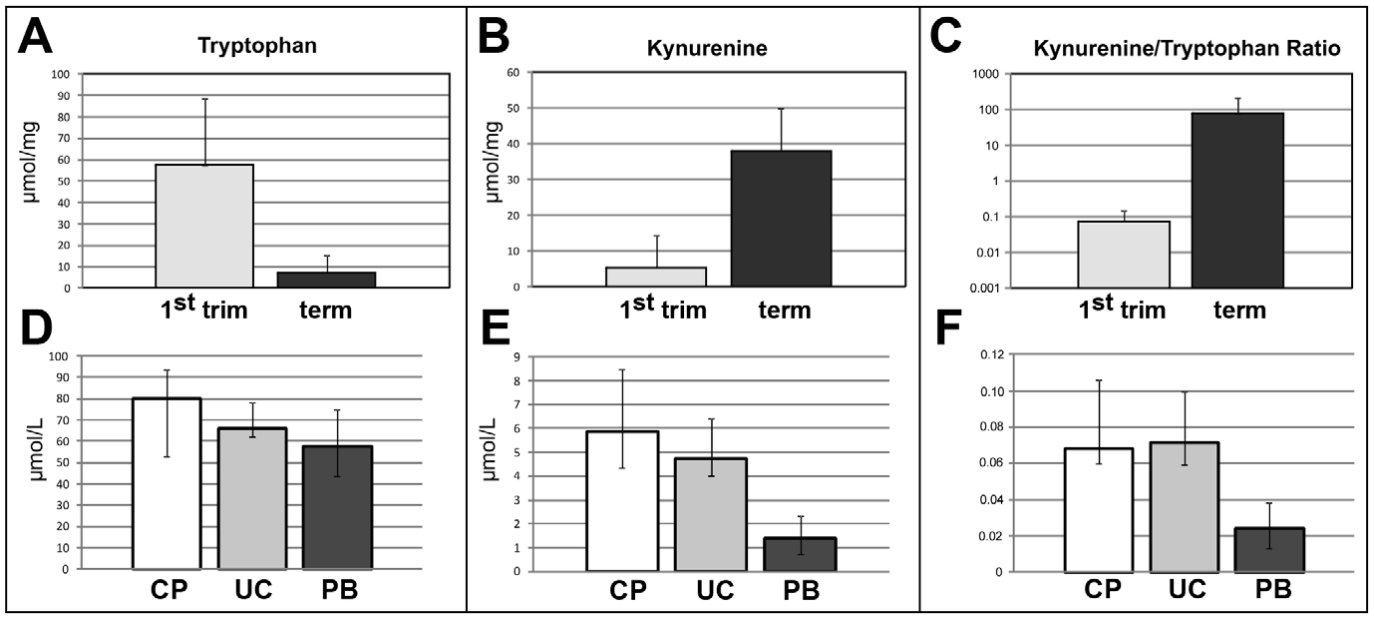
Chorionic tryptophan-degrading activity. Concentration of tryptophan (A, D), kynurenine (B, E) and the kyn/trp ratio (C, F) in placenta villous tissues (A, B, C) at first trimester (grey bars) and at term (black bars; indicated are the means+SD. For all three comparisons p<0.001) and in blood samples (D, E, F) from chorionic plate vessels of term placenta (CP, white bars), from umbilical cord blood (UC, light grey bars) and from peripheral blood of healthy blood donors (PB, dark grey bars). D–F: Indicated are the medians and the ranges. Assuming a significance level of 0.01, the test results for all three parameters are significant. The difference between the groups CP and PB is significant for all three parameters at p<0.001.

We confirmed the upregulation of IDO1 from first trimester to term placenta by measuring the levels of tryptophan and kynurenine and the Kyn/trp ratio in lysed tissues (Fig. 9a–c). In parallel to our immunohistochemical and RT-PCR results, tryptophan concentration measured in the tissue decreased (p<0.001) from first trimester villi (57.4+31.13 µmol/mg, mean+standard deviation) to term villi (7.2+8.14 µmol/mg, Fig. 9a), the concentration of kynurenine increased (p<0.001) from first trimester (5.3+8.94 µmol/mg) to term villi (38.0+11.97 µmol/mg, Fig. 9b). So the mean kynurenine/tryptophan ratio, allowing for an estimation of antecedent IDO activity of placental tissue increases about thousand-fold (p<0.001) from first trimester (0.076+0.072) to term placenta (74.419+126.291) (Fig. 9c).

As endothelial IDO1 may be supposed to act upon the vessel content and differences in concentrations of tryptophan degradation products might be expected after delivery, i. e. after interruption of the blood circulation, we tested for the concentrations of tryptophan and kynurenine in various blood compartments (Fig. 9d–f). Concentrations of tryptophan were 80.2 µmol/L (median, range 52.9–93.8) in blood drawn from vessels of the chorionic plate (n = 8), 67.0 (62.4–78.4) µmol/L for umbilical cord blood (n = 9), and 57.7 (43.6–75.1) µmol/L for peripheral blood from adult persons (n = 38). We detected kynurenine at 5.9 µmol/L (median, range 4.3–8.5) in blood from vessels of the chorionic plate, at 4.7 (4.0–6.4) µmol/L in umbilical cord blood and at 1.4 (0.7–2.3) µmol/L in peripheral blood. The kynurenine/tryptophan ratio was 0.068 (median, range 0.0601–0.106) for blood from vessels of the chorionic plate, 0.0707 (0.0595–0.0995) for umbilical cord blood, and 0.0238 (median, range 0.0131–0.0384) for peripheral blood. Assuming a significance level of 0.01, the test results for all three parameters are significant. The exact results fully support the obtained asymptotic two-sided p-values from the Kruskal-Wallis tests. Thus, the null hypothesis of identical sample medians can be rejected throughout.

In order to avoid the problem of multiple testing, we confined the subsequent analysis to comparison of blood from the chorionic vessels to peripheral blood, resulting in significant mean rank differences for all three parameters (tryptophan, kynurenine and the kynurenine/tryptophan ratio) at the level of p<0.001.

## Discussion

**Figure 10:**
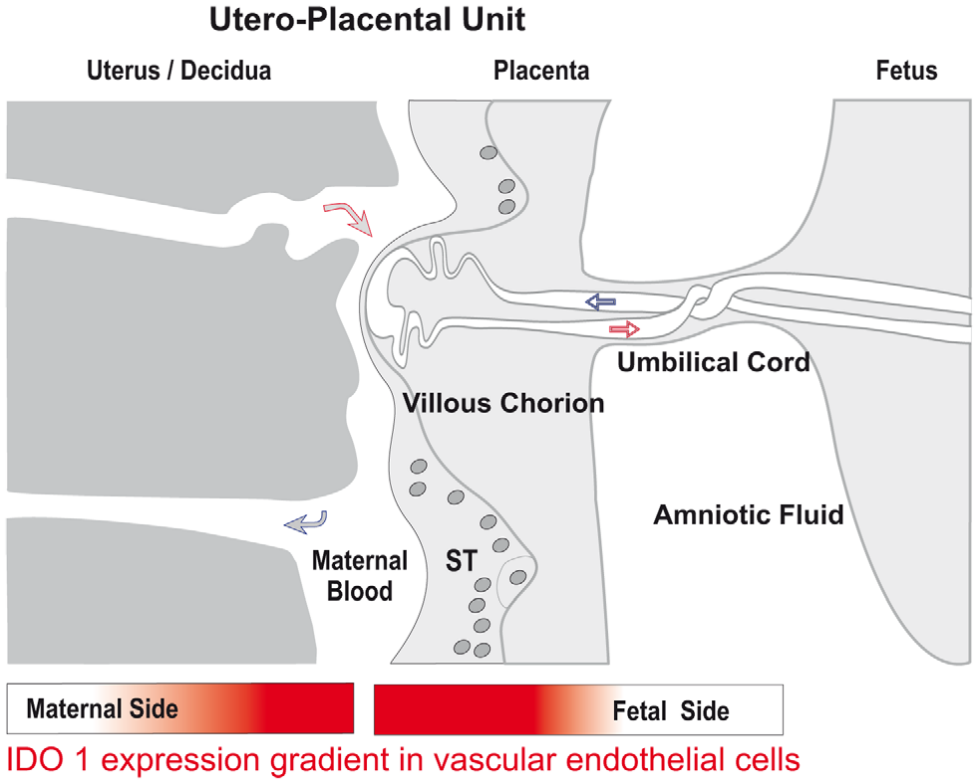
Illustration of the IDO1 expression gradient in vascular endothelial cells of fetal and maternal blood circulations at the utero-placental materno-fetal interface. The expression intensity increases close to the semi-allogeneic contact zone.

In extension of our previous observation of expression of IDO1 in the vascular endothelium of the decidua and the chorionic villi [7], we here report findings regarding the distribution of expression of IDO1 in the vasculature of the placenta. We found expression of IDO1 in endothelial cells of the villous chorion and chorionic tryptophan degrading activity increase during the course of pregnancy. For both fetal and maternal vascular systems we show an gradient of endothelial IDO1 expression towards to the feto-maternal contact zone in the human placenta (Fig. 10). Furthermore, in parallel with the stage of gestation this expression extends to locations increasingly distant from the interface (which is the case for the fetal side to a higher degree than for the maternal one). We see this phenomenon in context with the concentration of kynurenine measured in chorionic villous tissue, which increases with the stage of gestation, and also with the difference in the kyn/trp ratio in blood taken from the chorionic plate after delivery in contrast to adult blood.

**Figure 11:**
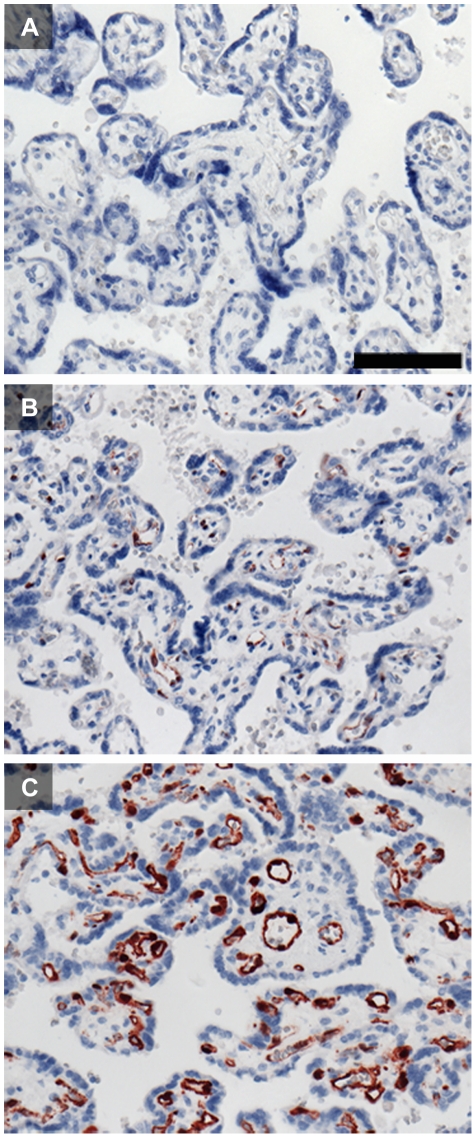
Influence of heat induced epitope retrieval (HIER) on IDO1 staining of formalin-fixed paraffin-embedded term placenta sections. Staining without any pre-treatment (A) does not allow for detection of IDO1. IDO1 can be localized in endothelial cells of the villous chorion after HIER using citrate buffer at pH 6 (B). HIER with retrieval buffer at pH 9 (C) enhanced the staining intensity and allows for a more detailed interpretation of IDO1 immunolocalization. The scale bar represents 100 µm.

There is considerable discrepancy among published reports on IDO1 expression in the human placenta. Causes accounting for this may include the variety of antibodies, concentrations and detection systems which have been used. The antigen or epitope retrieval may also affect the results. In Fig. 11 we show the impact of modification of just the single parameter of antigen retrieval on the outcome of immunohistological staining for IDO1 (comparison of the results obtained on formalin-fixed paraffin sections after heat induced epitope retrieval (HIER) with buffers at pH 6 and pH 9 and without any antigen retrieval). We obtained best results using HIER at pH 9; this was also shown for many other antibodies [26].

In contrast to some earlier reports including ours, we find IDO1 neither in villous nor in extravillous trophoblast [4], [5], [6], [7]. Earlier, we had used cryosections of the placenta for a Labelled (Strept-)Avidin-Biotin- (LSAB) technique [7]. In combination with a far more sensitive polymer-based detection system, the present report is based on paraffin sections allowing for better immunolocalization due to elimination of antigen carry-over. We argue that further descriptions of IDO1 expression by the trophoblast may be based on an unfortunate choice of the marker enzyme alkaline phosphatase [5], as placenta-specific endogenous alkaline phosphatase cannot be quenched sufficiently, and that IDO1-positive EC which underlie the trophoblast layer closely may have contaminated isolated trophoblasts in a study finding IDO1 mRNA in these cells [27]. A reported preferential staining of the invasive extravillous cytotrophoblast may be based on non-specific binding effects of an antibody to cryosections fixed with combination of methanol and formalin, or to using the same marker enzyme twice for immunohistochemical double-staining [4]. Immunohistological evidence provided by Ligam et al. does not allow for the conclusion of a localization of IDO1 to Hofbauer cells [6]. Expression of IDO1 by CD14-positive cells in the decidua could be ruled out in an earlier study [28]. Jones et al. characterized CD34-negative mesenchymal stem cells isolated from the human placenta which suppress allogeneic T cell proliferation [29]. Our immunohistology showed chorionic cells expressing IDO1 exclusively co-localizing with CD34. However, we cannot exclude that CD34-negative cells isolated from the placenta be induced in culture to express IDO1.

Our data leave us to conclude that tryptophan-degrading activity in villous tissue (which increases massively from first trimester to term in parallel to the vascular IDO1 expression) is exclusively due to vascular endothelial expression of IDO1.

Vascular endothelial cells in chorionic villi do not co-express MHC class II. This finding corresponds to a report of constitutive IDO expression in the epididymis and the placenta in both C57Bl/6 wild-type and IFN-gamma^−/−^ mice, suggesting a distinct regulatory mechanism for IDO1 induction in contrast to the well-known up-regulation by IFN-gamma [30]. On the other hand, we do see expression of HLA-DR in vessels of the decidua, but this is mainly confined to the endothelium of veins, whereas IDO1 expression in decidual endothelium is generally restricted to arteries. Thus, it seems unlikely that IDO1 expression at this location is related to secretion of IFN-gamma by the dense population of decidua NK cells.

What may be the biological role of vascular endothelial IDO1 in the vicinity of the feto-maternal interface?

It is reported that IDO-activity in cytokine-stimulated HUVECs (but less so endothelial cells of other origin such as the saphenous vein or internal mammary or radial arteries) suppresses allogeneic T-cell responses. Transfection of HUVECs with IDO1 induces anergy in allospecific T-cells which can also act as regulatory cells [31]. In allotransplants tolerance was mediated by regulatory T cell and IFN-gamma inducing the expression of IDO1 in EC of the graft [32].

We may assume that the feto-maternal border, the villous trophoblast layer, is not always perfectly impervious. This is clearly not the case in the course of an intervention such as a chorionic villous biopsy, but may also happen to some extent in a normal pregancy, e. g. due to intense active movement of the fetus or passive trauma from outside. In these cases an exchange of a small amount of fetal and maternal blood is possible, which means that immunocompetent lymphocytes come into contact with the endothelium of the other organism. For both hemiallogeneic organisms, suppression of an allogeneic immune response will be mandatory in these cases. It is established that the fetal immune system has the ability to respond to antigens as early as by 18 weeks of gestation [33]. A limited transfer of cells from the mother to the fetus and vice versa is the basis for the well recognized phenomenon of microchimerism [34], [35]. Fetal Y-chromosome DNA was detected in the maternal circulation in cellular compartments beginning at 7 to 16 weeks [36], [37]. On the other hand, while blood exchange peaks at the perinatal stage, prenatal transfer of nucleated maternal cells into the fetal circulation was demonstrated to occur as early as the 13^th^ week of gestation [38]. This finding parallels the fact that, although antiretroviral therapy for preventing mother-to-child transmission of HIV is partially effective when given only at parturition, the efficacy is greater when given throughout pregnancy [39]. Suppression of an allogeneic immune response will be even more important as endothelial cells function as semi-professional antigen-presenting cells [40].

Vascular endothelial expression of IDO1 is not restricted to the feto-maternal interface. There are positive microvascular endothelial cells in a variety of tumors [41], [42] (and own unpublished observations for hepatocellular carcinoma), leaving room for speculation on analogies of immune suppression in the placenta and suppression of the immune response to tumors.

A hint concerning a possible non-immunological role of endothelial expression of IDO1 has be given by the recent report that kynurenine metabolized by IDO which is expressed in the endothelium of mice infected with malaria parasites or induced by endotoxinemia contributes to arterial vessel relaxation and the control of blood pressure [43]. We found high concentrations of kynurenine in the serum or plasma from the umbilical cord or from vessels of the chorionic plate. These concentrations were much higher than in plasma samples of peripheral blood in pregnant or non-pregnant women [44], [45] or in blood from healthy blood donors [19] and are probably due to IDO1 activity of the endothelial cells. This suggests that endothelial IDO1 expressed in healthy placenta and decidua play a role in keeping vessels dilated which are vital for the maintenance of the fetus who in the course of pregnancy is increasingly dependent on maternal blood circulation. Given the relatively high concentration of kynurenine in the umbilical cord blood, this might provide a systemic effect on fetal circulation. For the circulation of the pregnant women no such systemic effect is to be expected, as the kynurenine concentration in the peripheral blood remains low during pregnancy [44], [45]. However, uterine expression of endothelial IDO1 may lead to local vasodilatation.

Reduced placenta IDO activity correlates with the severity of pre-eclampsia. Pregnant mice carrying hemiallogeneic concepti treated with an IDO inhibitor developed high blood pressure and proteinuria in addition to local circulation impairment in the placenta, which is analogous to the lesions that are characteristic of human pre-eclampsia [46]. It remains to be elucidated whether this is a direct effect based on blocking of endothelial IDO.

One might argue for a further potential role of IDO1: The enzyme is implicated in inhiting growth of intracellular parasites (Toxoplasma gondii, Chlamydia psittaci) as well as extracellular bacteria [18]. IDO1 expression in endothelial cells may inhibit extravasation of microbial pathogens from the blood into the tissue through the depletion of tryptophan in the local tissue adjacent to the blood stream [30]. This may contribute to protection of the fetus against infection.

## References

[1] Munn DH, Shafizadeh E, Attwood JT, Bondarev I, Pashine A (1999). Inhibition of T cell proliferation by macrophage tryptophan catabolism.. J Exp Med.

[2] Munn DH, Zhou M, Attwood JT, Bondarev I, Conway SJ (1998). Prevention of allogeneic fetal rejection by tryptophan catabolism [see comments].. Science.

[3] Koch CA, Platt JL (2007). T cell recognition and immunity in the fetus and mother.. Cell Immunol.

[4] Hönig A, Rieger L, Kapp M, Sutterlin M, Dietl J (2004). Indoleamine 2,3-dioxygenase (IDO) expression in invasive extravillous trophoblast supports role of the enzyme for materno-fetal tolerance.. J Reprod Immunol.

[5] Kudo Y, Boyd CA, Spyropoulou I, Redman CW, Takikawa O (2004). Indoleamine 2,3-dioxygenase: distribution and function in the developing human placenta.. J Reprod Immunol.

[6] Ligam P, Manuelpillai U, Wallace EM, Walker D (2005). Localisation of indoleamine 2,3-dioxygenase and kynurenine hydroxylase in the human placenta and decidua: implications for role of the kynurenine pathway in pregnancy.. Placenta.

[7] Sedlmayr P, Blaschitz A, Wintersteiger R, Semlitsch M, Hammer A (2002). Localization of indoleamine 2,3-dioxygenase in human female reproductive organs and the placenta.. Mol Hum Reprod.

[8] Munn DH, Sharma MD, Hou D, Baban B, Lee JR (2004). Expression of indoleamine 2,3-dioxygenase by plasmacytoid dendritic cells in tumor-draining lymph nodes.. J Clin Invest.

[9] Adams O, Besken K, Oberdorfer C, MacKenzie CR, Russing D (2004). Inhibition of human herpes simplex virus type 2 by interferon gamma and tumor necrosis factor alpha is mediated by indoleamine 2,3-dioxygenase.. Microbes Infect.

[10] MacKenzie CR, Heseler K, Muller A, Daubener W (2007). Role of indoleamine 2,3-dioxygenase in antimicrobial defence and immuno-regulation: tryptophan depletion versus production of toxic kynurenines.. Curr Drug Metab.

[11] Suzuki S, Tone S, Takikawa O, Kubo T, Kohno I (2001). Expression of indoleamine 2,3-dioxygenase and tryptophan 2,3-dioxygenase in early concepti.. Biochem J.

[12] Forouhar F, Anderson JL, Mowat CG, Vorobiev SM, Hussain A (2007). Molecular insights into substrate recognition and catalysis by tryptophan 2,3-dioxygenase.. Proc Natl Acad Sci U S A.

[13] Ball HJ, Yuasa HJ, Austin CJ, Weiser S, Hunt NH (2008). Indoleamine 2,3-dioxygenase-2; a new enzyme in the kynurenine pathway.. Int J Biochem Cell Biol.

[14] Löb S, Konigsrainer A, Schafer R, Rammensee HG, Opelz G (2008). Levo- but not dextro-1-methyl tryptophan abrogates the IDO activity of human dendritic cells.. Blood.

[15] Löb S, Konigsrainer A, Zieker D, Brucher BL, Rammensee HG (2009). IDO1 and IDO2 are expressed in human tumors: levo- but not dextro-1-methyl tryptophan inhibits tryptophan catabolism.. Cancer Immunol Immunother.

[16] Metz R, Duhadaway JB, Kamasani U, Laury-Kleintop L, Muller AJ (2007). Novel tryptophan catabolic enzyme IDO2 is the preferred biochemical target of the antitumor indoleamine 2,3-dioxygenase inhibitory compound D-1-methyl-tryptophan.. Cancer Res.

[17] Yuasa HJ, Takubo M, Takahashi A, Hasegawa T, Noma H (2007). Evolution of vertebrate indoleamine 2,3-dioxygenases.. J Mol Evol.

[18] Däubener W, Schmidt SK, Heseler K, Spekker KH, MacKenzie CR (2009). Antimicrobial and immunoregulatory effector mechanisms in human endothelial cells. Indoleamine 2,3-dioxygenase versus inducible nitric oxide synthase.. Thromb Haemost.

[19] Kositz C, Schroecksnadel K, Grander G, Schennach H, Kofler H (2008). High serum tryptophan concentration in pollinosis patients is associated with unresponsiveness to pollen extract therapy.. Int Arch Allergy Immunol.

[20] Lang I, Schweizer A, Hiden U, Ghaffari-Tabrizi N, Hagendorfer G (2008). Human fetal placental endothelial cells have a mature arterial and a juvenile venous phenotype with adipogenic and osteogenic differentiation potential.. Differentiation.

[21] Takikawa O, Kuroiwa T, Yamazaki F, Kido R (1988). Mechanism of interferon-gamma action. Characterization of indoleamine 2,3-dioxygenase in cultured human cells induced by interferon-gamma and evaluation of the enzyme-mediated tryptophan degradation in its anticellular activity.. J Biol Chem.

[22] Blaschitz A, Hutter H, Leitner V, Pilz S, Wintersteiger R (2000). Reaction patterns of monoclonal antibodies to HLA-G in human tissues and on cell lines: a comparative study.. Hum Immunol.

[23] Blaschitz A, Juch H, Volz A, Hutter H, Daxboeck C (2005). The soluble pool of HLA-G produced by human trophoblasts does not include detectable levels of the intron 4-containing HLA-G5 and HLA-G6 isoforms.. Mol Hum Reprod.

[24] Blaschitz A, Gauster M, Dohr G (2008). Application of cryo-compatible antibodies to human placenta paraffin sections.. Histochem Cell Biol.

[25] Widner B, Werner ER, Schennach H, Wachter H, Fuchs D (1997). Simultaneous measurement of serum tryptophan and kynurenine by HPLC.. Clin Chem.

[26] Yamashita S (2007). Heat-induced antigen retrieval: mechanisms and application to histochemistry.. Prog Histochem Cytochem.

[27] Dong M, Ding G, Zhou J, Wang H, Zhao Y (2008). The effect of trophoblasts on T lymphocytes: possible regulatory effector molecules--a proteomic analysis.. Cell Physiol Biochem.

[28] Cupurdija K, Azzola D, Hainz U, Gratchev A, Heitger A (2004). Macrophages of human first trimester decidua express markers associated to alternative activation.. Am J Reprod Immunol.

[29] Jones BJ, Brooke G, Atkinson K, McTaggart SJ (2007). Immunosuppression by placental indoleamine 2,3-dioxygenase: a role for mesenchymal stem cells.. Placenta.

[30] Hansen AM, Ball HJ, Mitchell AJ, Miu J, Takikawa O (2004). Increased expression of indoleamine 2,3-dioxygenase in murine malaria infection is predominantly localised to the vascular endothelium.. Int J Parasitol.

[31] Beutelspacher SC, Tan PH, McClure MO, Larkin DF, Lechler RI (2006). Expression of indoleamine 2,3-dioxygenase (IDO) by endothelial cells: implications for the control of alloresponses.. Am J Transplant.

[32] Thebault P, Condamine T, Heslan M, Hill M, Bernard I (2007). Role of IFNgamma in allograft tolerance mediated by CD4+CD25+ regulatory T cells by induction of IDO in endothelial cells.. Am J Transplant.

[33] Holt PG, Jones CA (2000). The development of the immune system during pregnancy and early life.. Allergy.

[34] Nelson JL (2008). Your cells are my cells.. Sci Am.

[35] Vernochet C, Caucheteux SM, Kanellopoulos-Langevin C (2007). Bi-directional cell trafficking between mother and fetus in mouse placenta.. Placenta.

[36] Ariga H, Ohto H, Busch MP, Imamura S, Watson R (2001). Kinetics of fetal cellular and cell-free DNA in the maternal circulation during and after pregnancy: implications for noninvasive prenatal diagnosis.. Transfusion.

[37] Krabchi K, Gros-Louis F, Yan J, Bronsard M, Masse J (2001). Quantification of all fetal nucleated cells in maternal blood between the 18th and 22nd weeks of pregnancy using molecular cytogenetic techniques.. Clin Genet.

[38] Lo ES, Lo YM, Hjelm NM, Thilaganathan B (1998). Transfer of nucleated maternal cells into fetal circulation during the second trimester of pregnancy.. Br J Haematol.

[39] Lallemant M, Jourdain G, Le Coeur S, Kim S, Koetsawang S (2000). A trial of shortened zidovudine regimens to prevent mother-to-child transmission of human immunodeficiency virus type 1. Perinatal HIV Prevention Trial (Thailand) Investigators.. N Engl J Med.

[40] Knolle PA (2006). Cognate interaction between endothelial cells and T cells.. Results Probl Cell Differ.

[41] Riesenberg R, Weiler C, Spring O, Eder M, Buchner A (2007). Expression of indoleamine 2,3-dioxygenase in tumor endothelial cells correlates with long-term survival of patients with renal cell carcinoma.. Clin Cancer Res.

[42] Batista CE, Juhasz C, Muzik O, Kupsky WJ, Barger G (2009). Imaging correlates of differential expression of indoleamine 2,3-dioxygenase in human brain tumors.. Mol Imaging Biol.

[43] Wang Y, Liu H, McKenzie G, Witting PK, Stasch JP (2010). Kynurenine is an endothelium-derived relaxing factor produced during inflammation.. Nat Med.

[44] Schröcksnadel H, Baier-Bitterlich G, Dapunt O, Wachter H, Fuchs D (1996). Decreased plasma tryptophan in pregnancy.. Obstet Gynecol.

[45] Schröcksnadel K, Widner B, Bergant A, Neurauter G, Schennach H (2003). Longitudinal study of tryptophan degradation during and after pregnancy.. Life Sci.

[46] Nishizawa H, Hasegawa K, Suzuki M, Achiwa Y, Kato T (2008). Mouse model for allogeneic immune reaction against fetus recapitulates human pre-eclampsia.. J Obstet Gynaecol Res.

